# Effect of combined therapy with catheter-directed thrombolysis and factor Xa inhibitor for inferior vena cava thrombosis: A case report

**DOI:** 10.1097/MD.0000000000011221

**Published:** 2018-07-13

**Authors:** Daishi Nonaka, Hiroyuki Takase, Masashi Machii, Kazuto Ohno

**Affiliations:** Department of Internal Medicine, Enshu Hospital, JA Shizuoka Kohseiren, Chuo, Naka-ku, Hamamatsu, Shizuoka, Japan.

**Keywords:** catheter-directed thrombolysis, factor Xa inhibitor, inferior vena cava thrombosis

## Abstract

**Rationale:**

Inferior vena cava (IVC) thrombosis is an under-recognized entity that is associated with a mortality rate approaching twice that of lower extremity deep venous thrombosis (DVT). Thrombolytic therapy not only results in greater lysis, but also results in higher complication rates than anticoagulation alone. Catheter-directed thrombolysis (CDT), which is effective in accomplishing local resolution whilst reducing bleeding complications, has been established as an alternative treatment for patients with extensive DVT.

**Patient concerns:**

We report the case of a 70-year-old man who was admitted due to warmness, pain, and swelling in his left leg and a feeling of gait disturbance.

**Diagnoses:**

Contrast-enhanced computed tomography and venous ultrasonography revealed a pulmonary embolism and extensive DVT spreading to the IVC.

**Interventions:**

First, the patient was treated with fondaparinux. Since this was inadequate, he underwent CDT using a Fountain infusion catheter. Then, CDT was switched to direct oral anticoagulant (DOAC) treatment.

**Outcomes:**

Both CDT and subsequent DOAC treatments dramatically improved the DVT. His subjective symptoms have disappeared, and no recurrence of thrombosis has been identified.

**Lessons:**

The present case showed the therapeutic effect of CDT, which preceded DOAC treatment, on an extensive DVT.

## 1 Introduction

Venous thromboembolism (VTE), which encompasses deep venous thrombosis (DVT) and pulmonary embolism (PE), is a serious disorder with major potential complications (including recurrent VTE, postthrombotic syndrome [PTS] and sudden death).^[1]^ Although DVT is the third most common cardiovascular disease following acute myocardial infarction and stroke,^[2]^ massive DVT extending to the inferior vena cava (IVC) is rare. Early extensive thrombosis with a high risk of PE is an indicator for interventional treatment. According to the current evidence, catheter-directed thrombolysis (CDT) can reduce a clot burden and recurrence of DVT, and consequently prevent the formation of PTS compared with systemic anticoagulation.^[3]^ The present report describes the case of a patient with a PE and massive DVT spreading to the IVC who was treated with both CDT and a factor Xa inhibitor.

## 2 Case report

A 70-year-old man was referred to our hospital by his primary doctor because of warmness, pain, and swelling in his left leg and a feeling of gait disturbance 2 days previously. The patient had a history of bronchial asthma, which was diagnosed at 50 years of age. Oral steroids had been prescribed from 61 years of age, and he receives insulin treatment due to steroid-induced diabetes mellitus. He also has a medical history of eosinophilic sinusitis, eosinophilic pneumonia, and cerebral infarction.

On physical examination, he was 1.76 m tall and weighed 68.0 kg (body mass index = 22.0 kg/m^2^). There was swelling and tenderness in his left leg and the left thigh circumference was greater than the right (46.7 cm vs 43.0 cm). His blood pressure, pulse rate, respiratory rate, and arterial oxygen saturation were 151/83 mm Hg, 110 beats/min, 16/min, and 98% (room air), respectively. The main laboratory findings were as follows: D-dimer, 44.1 μg/mL (normal range, <1.0 μg/mL); C reactive protein, 7.17 mg/dL (0.00–0.47 mg/dL); HbA1c, 9.6% (4.6–6.2%); protein C, 35% (64–146%); and antithrombin III, 85% (97–111%). The patient's other laboratory data are shown in Table 1.

**Table 1:**
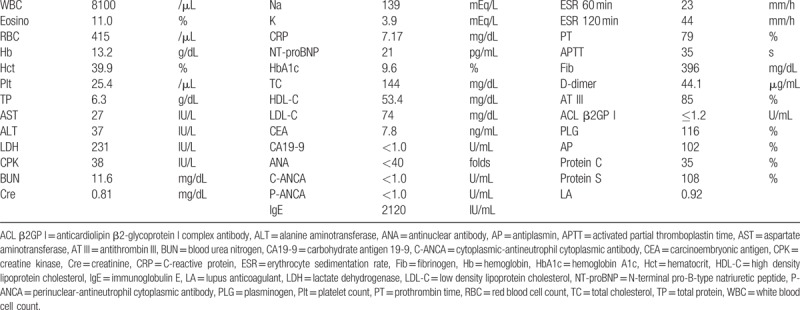
Patient's laboratory data on admission.

**Figure 1:**
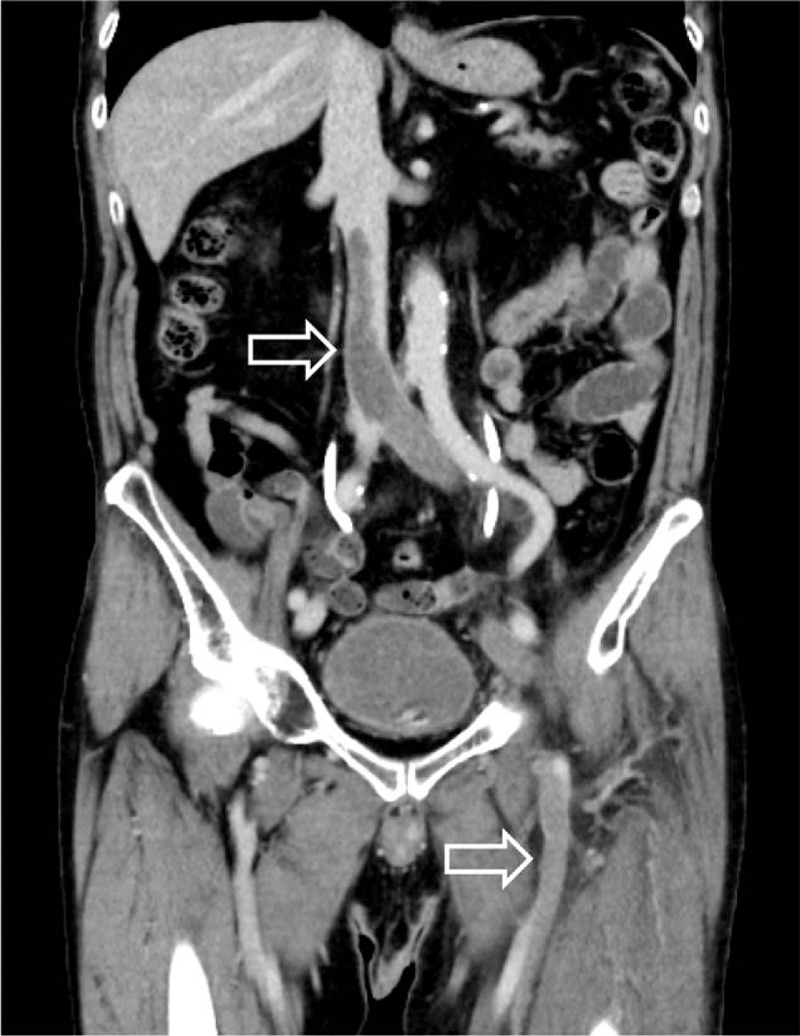
Abdominal computed tomography scan on admission showing a massive thrombus in the inferior vena cava and iliofemoral vein (arrow).

**Figure 2:**
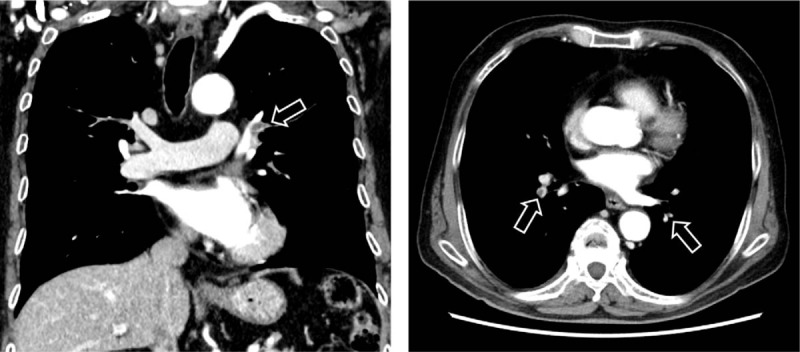
Chest computed tomography scan on admission showing a contrast deficit in the bilateral pulmonary artery (arrow).

Although the level of carcinoembryonic antigen as a tumor marker was increased (7.8 U/mL; 0.0–5.0 U/mL), malignancy was not observed on further examinations including computed tomography (CT). Electrocardiography exhibited sinus tachycardia and findings on the chest radiograph were normal. Venous ultrasonography showed extensive thrombosis in the left iliofemoral vein, left popliteal vein, and left posterior tibial vein. In addition to these thrombi, contrast-enhanced CT detected spreading of the thrombus in the IVC (Fig. 1), with several thrombi in the left upper lobe branch and the bilateral lower lobe branch (Fig. 2). According to these findings, he was diagnosed with PE and massive DVT.

**Figure 3:**
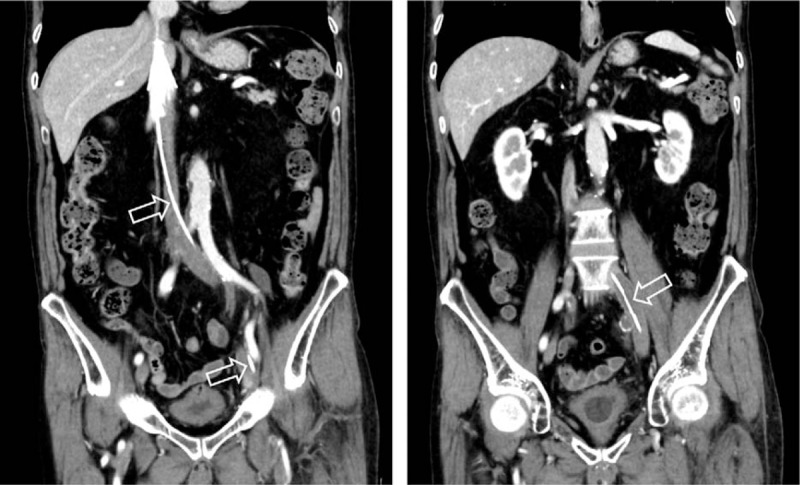
Abdominal computed tomography scan showing catheter-directed thrombolysis treatment using a Fountain infusion catheter (arrow).

**Figure 4:**
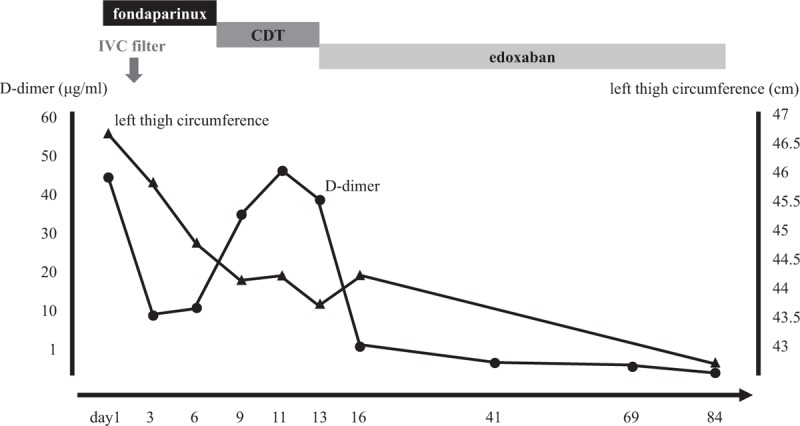
Clinical course of the patient with a pulmonary embolism and massive deep venous thrombosis. CDT = catheter-directed thrombolysis, IVC = inferior vena cava.

On the day of admission, subcutaneous fondaparinux (7.5 mg once daily) was started and a Günther Tulip Filter (Cook Medical Inc., Bloomington, Ind) was deployed in the suprarenal IVC via the right internal jugular vein to prevent a fatal PE. Because the DVT did not improve despite the reduction of the PE after 7 days of treatment with fondaparinux, CDT was initiated on the eighth day of hospitalization. Hydrophilic guidewire was inserted across the thrombus using Merit MAK mini access kits (Merit Medical Systems Inc., South Jordan, UT), via the left popliteal vein. A 50 cm-long Fountain infusion catheter (SHEEN MAN Co. Ltd, Osaka, Japan) was then passed through the entire extent of the thrombus in the venous segment (Fig. 3). Then, urokinase (240,000 IU/day) was repeatedly splashed out through the catheter in approximately 30 minutes, once a day for 6 days. In addition, continuous intravenous administration of unfractionated heparin was started to keep the activated partial thromboplastin time (APTT) 1.5- to 2.0-fold longer than the control value. After 6 days of CDT treatment, the pain and swelling in the patient's left leg improved, and the left thigh circumference reduced to 44.0 cm. However, because CT demonstrated residual thrombi from the IVC to the left posterior tibial vein, the thrombolytic treatment for DVT was switched from CDT to direct oral anticoagulant (edoxaban; 60 mg/day). The patient was discharged without bleeding and infection on the 17th day after hospitalization. The clinical course of this patient is shown in Fig. 4.

**Figure 5:**
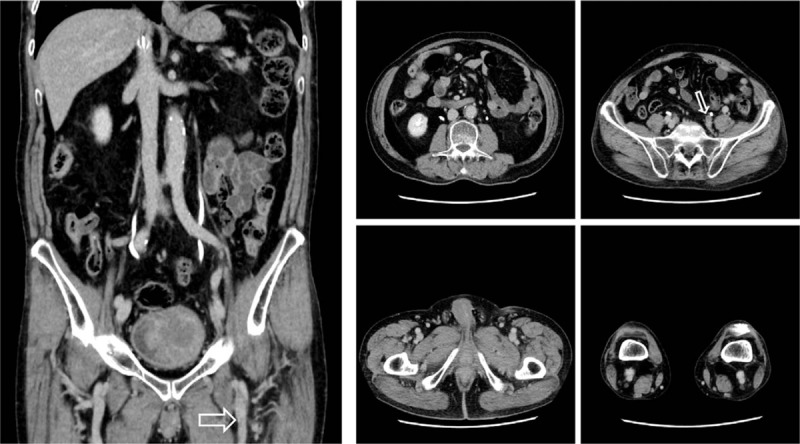
Abdominal computed tomography scan approximately 2 months after the initiation of treatment with both catheter-directed thrombolysis and factor Xa inhibitor. The arrows indicate reduced thrombosis.

Approximately 2 months after the start of oral medical treatment, there are few thrombi other than in part of the left iliac vein (Fig. 5), and D-dimer has decreased to 0.5 μg/mL. The thrombolytic therapy with edoxaban has been continued in this patient.

## 3 Discussion

We reported a case of a 70-year-old man with a massive DVT extending from the lower extremity to the IVC, which was effectively treated with CDT and a factor Xa inhibitor.

Medical management is generally the first line of treatment for a PE and DVT. We selected fondaparinux for anticoagulation at first, because we have previous experience of its use as a VTE treatment. Furthermore, since the hemodynamics of this patient were preserved and he has been under steroid treatment, we did not perform CDT first due to the risk of bleeding and infection. Although the effectiveness of once-daily subcutaneous fondaparinux on DVT patients has been reported previously,^[4]^ it could not reduce thrombosis 1 week after hospitalization in this case. Because massive DVT may cause PTS and this patient had no contraindications for thrombolysis such as bleeding diathesis, malignancy, and renal failure, we changed anticoagulation as a monotherapy to CDT. The CaVenT study of patients with iliofemoral DVT demonstrated that the addition of CDT to anticoagulant therapy significantly improved iliofemoral patency as well as reducing the frequency of PTS compared to standard therapy alone.^[5]^ The guidelines from the American College of Chest Physicians recommend that CDT should be used in patients with a life expectancy of more than a year, good functional status, extensive iliofemoral thrombosis, and who present soon after the onset of symptoms.^[3]^ In contrast to systemic administration of the agent, CDT can increase infusion times leading to a high incidence of partial thrombolysis. By using a Fountain infusion catheter which has multiple side holes for dispersion of a thrombolytic agent, urokinase can be administrated directly into the thrombus. The conceivable advantages of intrathrombus infusion are as follows: clot removal efficacy is enhanced by the ability to achieve an increased intrathrombus drug concentration, and the additional mechanical thrombus disruption by directly spraying the thrombolytic drug may further enhance pharmacological dissolution of the thrombus.^[6]^ In fact, because CDT preceded the medication treatment, edoxaban dramatically reduced the thrombus except for the part in the iliac vein in this case. The duration of future treatment with edoxaban has been a matter of debate. We consider that edoxaban therapy should be continued as long as possible, because recurrence of an extensive DVT may induce a massive PE which causes sudden death, and permanent IVC filters are associated with the increased risk of IVC thrombosis.^[7]^ We should consider whether the use of an IVC filter is appropriate or not by evaluating both the risk of PE and the benefit of inserting the filter. At first, a retrievable IVC filter was used in the present case, but we decided to indwell the filter permanently because of the time taken to treat with the pharmacological agent. According to a previous report describing the safety and efficacy of suprarenal IVC filters,^[8]^ it was necessary to implant the filter into the suprarenal IVC due to thrombus propagation in this case.

A previous study reported that the incidence of a thrombus in the IVC was only 3.3% in about 25,000 DVT cases.^[9]^ Thrombosis of the IVC was prevalent in 60% to 80% of patients with congenital venous anomalies, especially in cases of IVC hypoplasia or aplasia.^[10]^ An IVC thrombosis without congenital abnormalities is uncommon and it is usually a result of a predisposing hypercoagulable state as a consequence of malignancy, smoking, obesity, and hormonal replacement therapy. Indeed, although the current case did not have anatomical anomalies of the IVC, the patient has been managed with oral steroids for a long time. It is known that use of systemic glucocorticoids is associated with a great increase in VTE risk.^[11]^ A previous study showed that glucocorticoids significantly enhanced plasminogen activator inhibitor-1 synthesis by human adipose tissue.^[12]^ Brotman et al^[13]^ demonstrated that clotting factors and fibrinogen level increased in a healthy man treated with dexamethasone. Furthermore, in the present case, the past medical history indicated that he had received treatment for eosinophil-related disorders. Although it did not finally lead to the diagnosis of allergic granulomatosus angiitis, the rate of peripheral blood eosinophils had increased more than 60% before admission. During this hospitalization, 33.0% eosinophils (absolute eosinophil count 3234/μL) were also observed despite continuing treatment with steroids. Indeed, the relation between eosinophilia and DVT has been reported.^[14]^ The mechanisms of eosinophil-induced thrombus formation are thought to include endothelial cell damage and inactivation of thrombomodulin by eosinophil cationic protein and major basic protein. Cugno et al^[15]^ described that increased tissue factors were expressed in patients with hypereosinophilic disorders, which may contribute to the exacerbation of thrombosis.

We concluded that not only anticoagulation therapy, but CDT therapy before treatment with a factor Xa inhibitor, markedly improved a PE and extensive DVT.

## Author contributions

**Investigation:** Daishi Nonaka, Hiroyuki Takase, Masashi Machii, Kazuto Ohno.
